# Leprosy Screening Based on Artificial Intelligence: Development of a Cross-Platform App

**DOI:** 10.2196/23718

**Published:** 2021-04-07

**Authors:** Márcio Luís Moreira De Souza, Gabriel Ayres Lopes, Alexandre Castelo Branco, Jessica K Fairley, Lucia Alves De Oliveira Fraga

**Affiliations:** 1 Multicentre Biochemistry and Molecular Biology Program Federal University of Juiz de Fora Governador Valadares-MG Brazil; 2 Fellowship of PROEX Program/UFJF Federal University of Juiz de Fora Governador Valadares-MG Brazil; 3 Reference Center for Endemic Diseases and Special Programs (SMS/GV) Governador Valadares-MG Brazil; 4 Emory University School of Medicine Atlanta, GA United States

**Keywords:** leprosy, artificial intelligence, random forest, Python, R, apps, mHealth, shinyApp

## Abstract

**Background:**

According to the World Health Organization, achieving targets for control of leprosy by 2030 will require disease elimination and interruption of transmission at the national or regional level. India and Brazil have reported the highest leprosy burden in the last few decades, revealing the need for strategies and tools to help health professionals correctly manage and control the disease.

**Objective:**

The main objective of this study was to develop a cross-platform app for leprosy screening based on artificial intelligence (AI) with the goal of increasing accessibility of an accurate method of classifying leprosy treatment for health professionals, especially for communities further away from major diagnostic centers. Toward this end, we analyzed the quality of leprosy data in Brazil on the National Notifiable Diseases Information System (SINAN).

**Methods:**

Leprosy data were extracted from the SINAN database, carefully cleaned, and used to build AI decision models based on the random forest algorithm to predict operational classification in paucibacillary or multibacillary leprosy. We used Python programming language to extract and clean the data, and R programming language to train and test the AI model via cross-validation. To allow broad access, we deployed the final random forest classification model in a web app via shinyApp using data available from the Brazilian Institute of Geography and Statistics and the Department of Informatics of the Unified Health System.

**Results:**

We mapped the dispersion of leprosy incidence in Brazil from 2014 to 2018, and found a particularly high number of cases in central Brazil in 2014 that further increased in 2018 in the state of Mato Grosso. For some municipalities, up to 80% of cases showed some data discrepancy. Of a total of 21,047 discrepancies detected, the most common was “operational classification does not match the clinical form.” After data processing, we identified a total of 77,628 cases with missing data. The sensitivity and specificity of the AI model applied for the operational classification of leprosy was 93.97% and 87.09%, respectively.

**Conclusions:**

The proposed app was able to recognize patterns in leprosy cases registered in the SINAN database and to classify new patients with paucibacillary or multibacillary leprosy, thereby reducing the probability of incorrect assignment by health centers. The collection and notification of data on leprosy in Brazil seem to lack specific validation to increase the quality of the data for implementations via AI. The AI models implemented in this work had satisfactory accuracy across Brazilian states and could be a complementary diagnosis tool, especially in remote areas with few specialist physicians.

## Introduction

### Leprosy Background

Leprosy is an infectious disease caused by *Mycobacterium leprae* and *Mycobacterium lepromatosis*, which affects the skin and peripheral nerves, causing stigma and disabilities that limit community involvement and social engagement [1]. According to the World Health Organization (WHO) report in 2020, there were 208,619 new leprosy cases registered globally in 2018, with a prevalence rate corresponding to 0.2 in 10,000 individuals. Brazil reported 27,893 new cases in 2019 and is considered the country with the second highest number of new leprosy cases registered in the world. Moreover, Brazil reported the highest number of retreated cases (6887), followed by India (5332). In-depth analysis of retreatment to determine the reasons for treatment interruption would improve compliance, effective use of drugs, and, to some extent, prevent the emergence of drug resistance. A review of new cases detected in the three most highly endemic countries (India, Brazil, and Indonesia) indicated that the number of new cases, the proportion of child cases, and those associated with disability have barely changed in Brazil and Indonesia for the past 5 years. Therefore, there is a long way to go to achieve the following criteria for leprosy elimination: (1) confirmed absence of children with leprosy for 5 consecutive years and (2) confirmed absence of new leprosy cases for 10 years [2].

Currently, the conventional diagnosis of leprosy is typically based on clinical evaluation alone, especially when histopathological analysis is not available. The clinical diagnosis is based on cardinal signs such as the presence of skin lesions (often with loss of sensitivity), thickening of the nerves, and presence of the pathogen in a skin smear or histological tissue samples. Based on this information, classifications are applied to aid in the understanding and treatment of the disease [1,3].

The Madrid classification divides leprosy patients into the following four categories: indeterminate, tuberculoid, borderline, and lepromatous [3]. However, the WHO has also proposed an operational classification to facilitate fieldwork. Patients with up to five skin lesions are classified as having paucibacillary leprosy and those with more than five skin lesions are classified as having multibacillary leprosy [4].

Currently, multidrug therapy is the main treatment for leprosy, which is based on schemes supported by the operational classification. The Guidelines Development Group, established by the WHO in 2018, recommends the same regimen of three drugs (rifampicin, dapsone, and clofazimine) for all leprosy patients, with a 6-month duration for paucibacillary cases and a 12-month duration for multibacillary cases. Some evidence suggests a potential increase in the risk of relapse for patients with paucibacillary leprosy using the previous two-drug regimen [4]. Therefore, this three-drug regimen has the potential to reduce the consequences of misclassifying multibacillary cases as paucibacillary cases (based on lesion count) and the implementation advantages of using the same combination of three drugs for both types [4-6].

Laboratory diagnosis can help differentiate leprosy from other dermatological/neurological diseases, especially in cases of suspected recurrence, and determine an appropriate treatment duration. In these cases, microscopic examination of the dermal smear is the method most commonly used because it is easy to perform and is of low cost. The bacilloscopy index (BI) is negative (0) in the tuberculoid and indeterminate forms, is strongly positive in the lepromatous type, and reveals a variable result in borderline cases [3].

In 2020, the WHO outlined the goal to interrupt leprosy transmission at the national or regional level by 2030. However, to achieve this goal, it is necessary to routinely implement active case detection and contact tracing. Therefore, it is urgent to improve the tools used for an early and precise diagnosis of new cases [2].

### App Characteristics

Brazil uses the Sistema de Informação Nacional de Agravos de Notificação/National Notifiable Diseases Information System (SINAN) to deal with epidemiological aspects of diseases with compulsory notification. A leprosy-specific form has to be filled out for each confirmed case, which involves information about the patient, including the number of lesions and affected nerves, grade of physical disability, and demographic variables, among others. All of these data are stored in SINAN’s online database and are available for epidemiological studies [7-10].

The app proposed in this study was based on an in-depth analysis of the SINAN database using machine learning, a research area that focuses on how computers acquire knowledge from data. Machine learning can be subclassified into two general types: unsupervised learning and supervised learning. Unsupervised learning does not have a focus on a predictable output, as its main objective is to identify data patterns. By contrast, supervised learning focuses on an outcome such as determining if an animal in a picture is a cat or a dog [11]. For app development in this study, we used random forest (RF), as one of the most effective algorithms of supervised learning that was developed by Leo Breiman [12], to predict the operational classification (paucibacillary or multibacillary) of a given leprosy case. Our research team previously successfully used this algorithm to predict new cases of leprosy among household contacts using molecular and serological inputs [13].

There are some existing apps that were also designed to help diagnose neglected tropical diseases (NTDs). According to the WHO in 2018, an app was developed to facilitate the diagnosis of NTDs of the skin, including leprosy. This app allows health care workers and the public to obtain information about a specific disease, such as its clinical features, management, and geographical distribution, and provides a list of potential diagnoses. The training guide is now updated with recent information and has been translated into an easy-to-use interactive mobile app available free of charge on both Android and iOS platforms [14]. Recently, a new app that helps health care professionals diagnose leprosy was launched by the Federal University of São Paulo [15]. However, among the cited references, none describes the creation process of apps designed to control leprosy using artificial intelligence (AI) to predict the diagnosis, leaving an important gap in the field that this study aimed to address.

### Study Objective

Given the above background, the main objective of our study was to develop a cross-platform app for leprosy screening based on AI. This app was designed to recognize patterns in leprosy cases registered in the SINAN database and to classify new cases as paucibacillary or multibacillary, thereby reducing the probability of misclassification by the health center.

## Methods

### Overview

**Figure 1:**
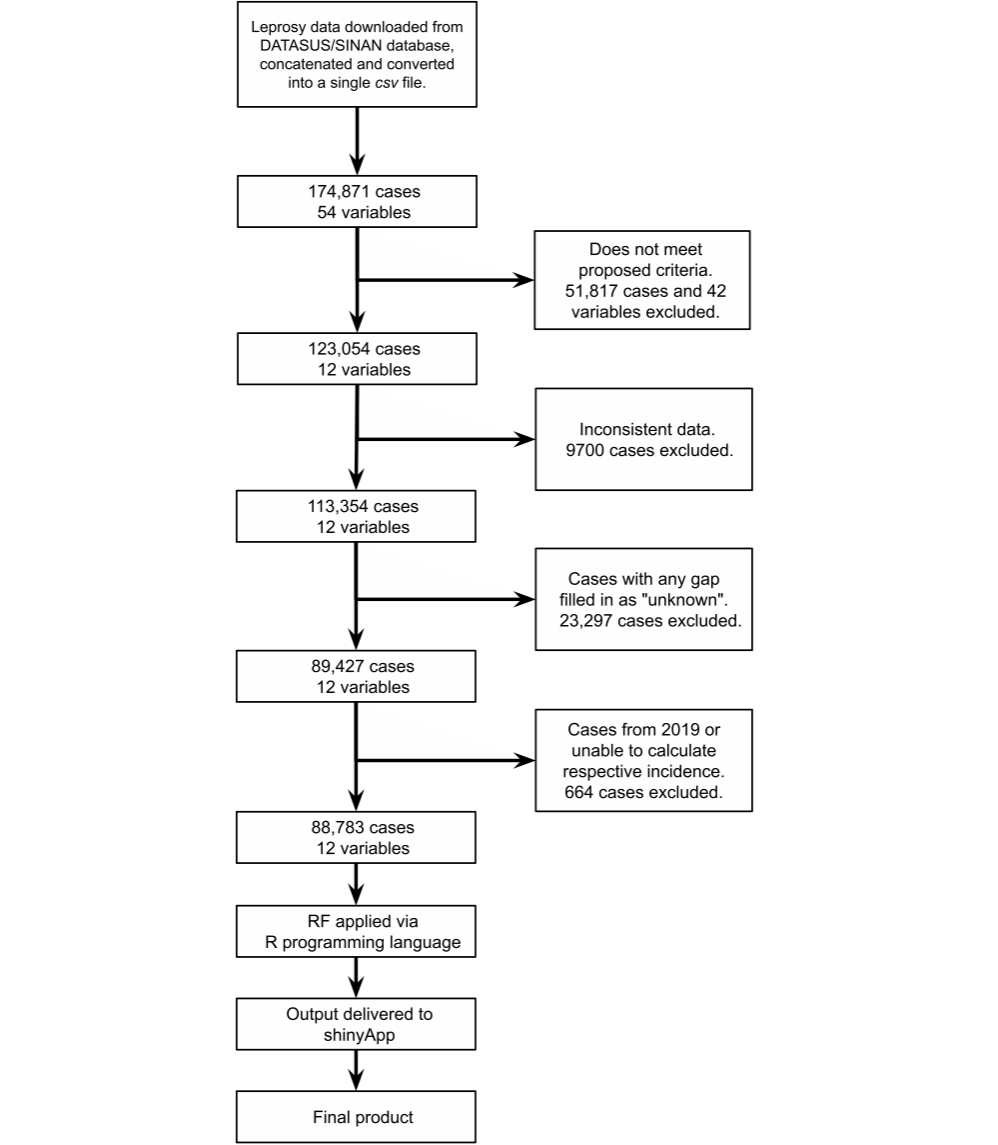
Flow diagram summarizing the data-processing and app-building steps. SINAN: Sistema de Informação Nacional de Agravos de Notificação (National Notifiable Diseases Information System); DATASUS: DATASUS: Sistema Único de Saúde (Unified Health System) data portal; RF: random forest; csv: Comma Separated Value.

We divided the stages of app construction into two steps: (1) processing raw data and obtaining a decision matrix, and (2) using the decision matrix to build the app for classifying a case given an input. An overview of the entire process is shown in Figure 1.

### Processing Raw Data

Initially, we downloaded all SINAN records related to leprosy cases from 2014 to 2019, which were converted to a single Comma Separated Values file. This procedure resulted in a 54-column file containing data on 174,871 cases, with each column corresponding to a specific variable reported by Brazilian health professionals about the leprosy cases notified.

Many of these columns (variables) were not relevant to our study. Therefore, we removed those that did not fulfill the criteria as shown in Table 1.

**Table 1:** Exclusion criteria and justifications.

Exclusion criteria	Justifications
Columns with more than 25,000 “NA” (not available)	The objective was to remove variables that many professionals have not declared the value of, as a large amount of missing data may impair processing. The number 25,000 was arbitrarily defined, focusing on not drastically reducing the total amount of data
Categorical variable with more than 53 input possibilities	R shows an alert when a categorical variable with more than 53 input possibilities is being used, given that the greater the number of input possibilities, the smaller the meaning of each input to the model
Variables that may induce a result	Some variables imply an operational classification (eg, “g-MB” therapeutic scheme implies that the patient has a case of multibacillary leprosy), causing bias to the model
Variables with no apparent correlation with the prediction.	Boruta [16], an algorithm to find the most relevant variables to predict outcomes in a given dataset, was used; variables with little relevance were excluded
Redundant variables	Redundant variables do not provide additional information to the model, and therefore there is no reason to keep both. An analysis using Python showed that some variables had almost 100% correspondence with another (eg, the state where the case was notified and the state where the patient lives). The Boruta algorithm is also useful to remove redundant variables.

The remaining dataset was composed of the variables age, gender, race, education, grade of disability, operational classification, BI, number of affected nerves, clinical form, municipality ID, number of household contacts, and the number of skin lesions. After this processing, we removed all lines with any entry of “NA” (not applicable), leaving a total of 123,054 cases.

The Brazilian Practical Guide on Leprosy was reviewed to define the following criteria to remove cases with any inconsistency: (i) samples with a positive BI are always cases of multibacillary leprosy; (ii) patients with paucibacillary leprosy should have five or fewer skin lesions; (iii) indeterminate and tuberculoid are always paucibacillary forms; (iv) borderline and lepromatous cases are always multibacillary forms; (v) indeterminate cases have no disability; and (vi) a maximum of approximately 18 nerve trunks are evaluated in clinical examinations [17]. After removing cases that were not consistent with these criteria, 113,354 cases remained. All lines with missing items (gender, race, schooling, grade of disability, operational classification, bacilloscopy, or clinical form) were removed to improve the accuracy of prediction, leaving 89,427 cases.

**Figure 2:**
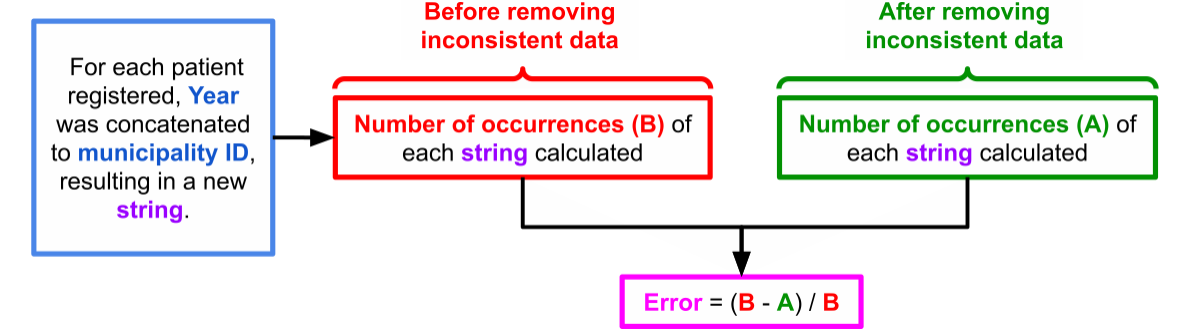
Method to calculate the error rate.

The Instituto Brasileiro de Geografia e Estatística (Brazilian Institute of Geography and Statistics), responsible for conducting the census, provides tables containing the estimated population by year and by municipality [18]. These tables were merged with all SINAN data (174,871 cases) to calculate the leprosy incidence by year and by city. However, it was necessary to delete the data for 2019 because of the lack of year-round records in SINAN, leaving 88,783 cases. Finally, comparing the number of cases after removing inconsistencies, we created an index that represents the percentage of patients with discrepancies in diagnosis (error) per year for each municipality, according to the workflow shown in Figure 2.

Confidence intervals of these municipality inconsistencies were calculated for each state of Brazil. We calculated the median of household contacts by city and year. All data processing up to this point was performed using Python 3.8 and WPS spreadsheets.

### Applying RF and Building the App

After initial processing with Python, the RF algorithm was applied to the resulting data using the R software package Random Forest. In addition to RF, there are several other machine-learning classification algorithms that could be appropriate for this task, such as naive Bayes [19], logistic regression [20], k-nearest neighbor [21], decision tree [22], and gradient boosting [23]. This list is not exhaustive, but includes the most common algorithms that were applied to our dataset for model comparison. The RF algorithm was ultimately chosen owing to its better performance, ease of use and interpretation, and wide dissemination in the literature. RF is an ensemble learning method for classification, regression, and other tasks, which operates by constructing a multitude of decision trees during training and then outputting the class that represents the mode of the categories (classification) or mean prediction (regression) of the individual trees [24].

After several tests to improve model accuracy, the following subset of variables was used to predict the operational classification of each case: region, state, city, age, number of skin lesions, affected nerves, household contacts, and bacilloscopy. Prediction of operational classification was chosen as the metric for evaluation instead of prediction of the clinical form for two main reasons: (1) Brazilian treatment is based on the operational classification [17], and (2) dichotomous models (in this case paucibacillary or multibacillary) have better results in RF-based analyses [25].

This multiplatform app was designed to meet the scientific demand for technological innovation concurrently with the lack of safe and accurate diagnoses in remote regions of Brazil, where training in clinical practice does not always match the international standards recommended by the WHO. In this sense, we incorporated only clinical variables reported by SINAN so that the app would be useful in the Sistema Único de Saúde (SUS; Unified Health System) throughout Brazil.

**Figure 3:**
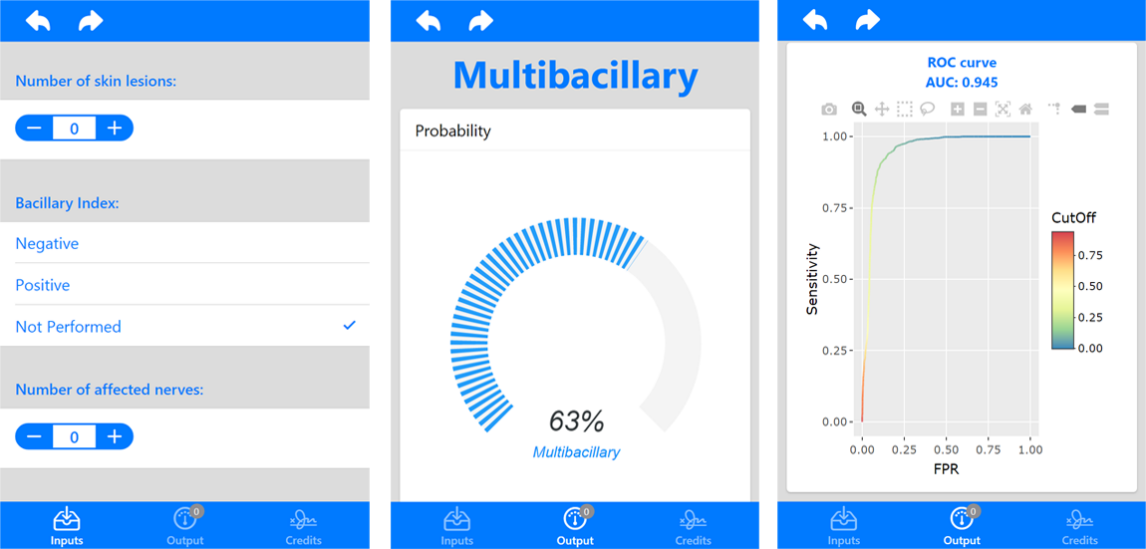
Screenshot representing the R ShinyApp input and output flows. The layout of the app may eventually change to improve user experience. ROC: receiver operating characteristic; AUC: area under the curve; FPR: false positive rate.

The decision forest obtained by the RF algorithm from the R package was deployed in a ShinyApp environment, which is a web service for constructing a friendly user interface. Figure 3 shows the structure and flow of the app. The receiver operating characteristic (ROC) curve [26] represents the true positive rate (TPR or sensitivity) as a function of the false positive rate (FPR or 1 – specificity). The colored scale on the right of the curve denotes the cutoffs distribution used to obtain a classification of multibacillary leprosy by the model. Each cutoff is related to one TPR and FPR point in the plot [27]. Given the number of true positives (TP), true negatives (TN), false positives (FP), and false negatives (FN), the TPR and FPR are defined as TP/TP+FN and FP/FP+TN, respectively.

A good model must enhance the TPR and decrease the FPR. Thus, the quality of the model may be represented by the area under the ROC curve (AUC) value [28]. The best models have an AUC close to 1 and the worst models have an AUC close to 0.

The value of each variable in the database has a different weight for the model. Some values approximate a paucibacillary classification, whereas other values approximate toward a multibacillary classification. Representing the distance between paucibacillary and multibacillary as a scale from 0 to 1, we can choose different values on this scale as the limit between the two classifications. These possible values are represented by the colored scale to the right of the ROC curve in Figure 3.

## Results

### Database Processing

#### Inconsistencies

Table 2 shows the 21,053 inconsistencies obtained in the dataset from a total of 174,871 individuals in the SINAN database in the period of 2014-2018. It is important to note that an individual might have more than one discrepancy. The main inconsistency identified was “operational classification does not match the clinical form.” This means that a lepromatous or borderline patient was misclassified as a paucibacillary patient, or that an indeterminate or tuberculoid patient was misclassified as a multibacillary patient.

**Table 2:** Number of occurrences per inconsistency.

Inconsistency	Number of occurrences
Operational classification does not match the clinical form	8545
Indeterminate with disability	4867
Indeterminate with affected nerves	3785
Paucibacillary with positive bacilloscopy	2825
Paucibacillary with more than 5 skin lesions	938
Patients with more than 18 affected nerves	93

#### Missing Data

Before data processing, the SINAN database had 35,616 lines with at least one NA, 26,539 lines with at least one unknown item (gender, race, schooling, grade of disability, operational classification, bacilloscopy, or clinical form), and 15,473 lines with both NA and an unknown item. There were a total of 77,628 cases with missing data, accounting for 44.39% of the total 174,871 cases. 

### Leprosy in Brazil

**Figure 4:**
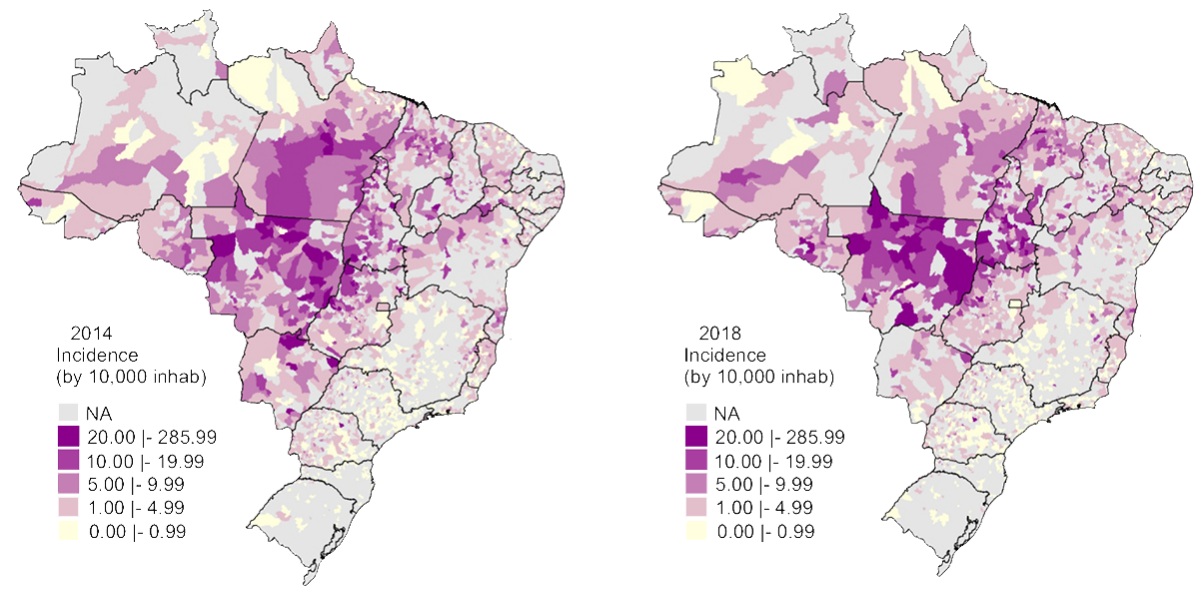
Geographic distribution of new annual cases of leprosy in Brazilian municipalities. inhab: inhabitants; NA: not available.

Based on available data in SUS, after cleaning the dataset, it was possible to geographically visualize the dispersion of leprosy incidence in Brazil over the period from 2014 to 2018 (Figure 4). A high number of cases were notably located in central Brazil. This number became even higher in 2018, highlighting the State of Mato Grosso with the highest number of leprosy case reports in the country. Some municipalities had an incidence of up to 285 cases per 10,000 inhabitants.

**Figure 5:**
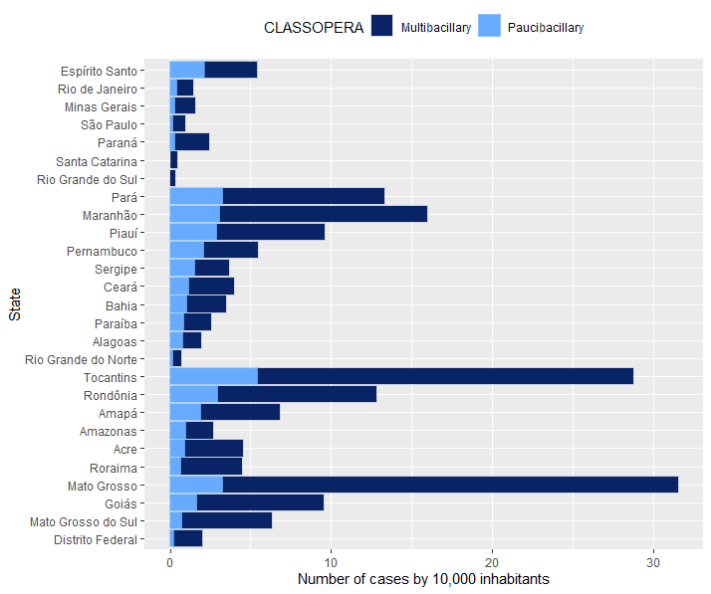
Distribution of leprosy cases in the Brazilian states from 2014 to 2018.

In addition to showing the geographical extent of the disease, the cleaned database included individuals from 4 to 106 years old (mean of 44 years), with an average of seven lesions, two affected nerves, and three household contacts per case. The BI was not calculated in 37.00% (32,853/88,783) of cases, and for the remaining cases, 41.18% (23,034/55,930) of the BI results were positive. In total, the database reported multibacillary leprosy in 76.66% (68,061/88,783) of patients, who were scattered throughout the Brazilian territory (Figure 5).

Figure 5 shows the distribution of paucibacillary and multibacillary cases among Brazilian states (per 10,000 inhabitants) with the highest numbers found in Mato Grosso (31.5), Tocantins (28.8), Maranhão (15.9), Pará (13.3), and Rondônia (12.8), which are all states with a predominance of multibacillary cases.

**Figure 6:**
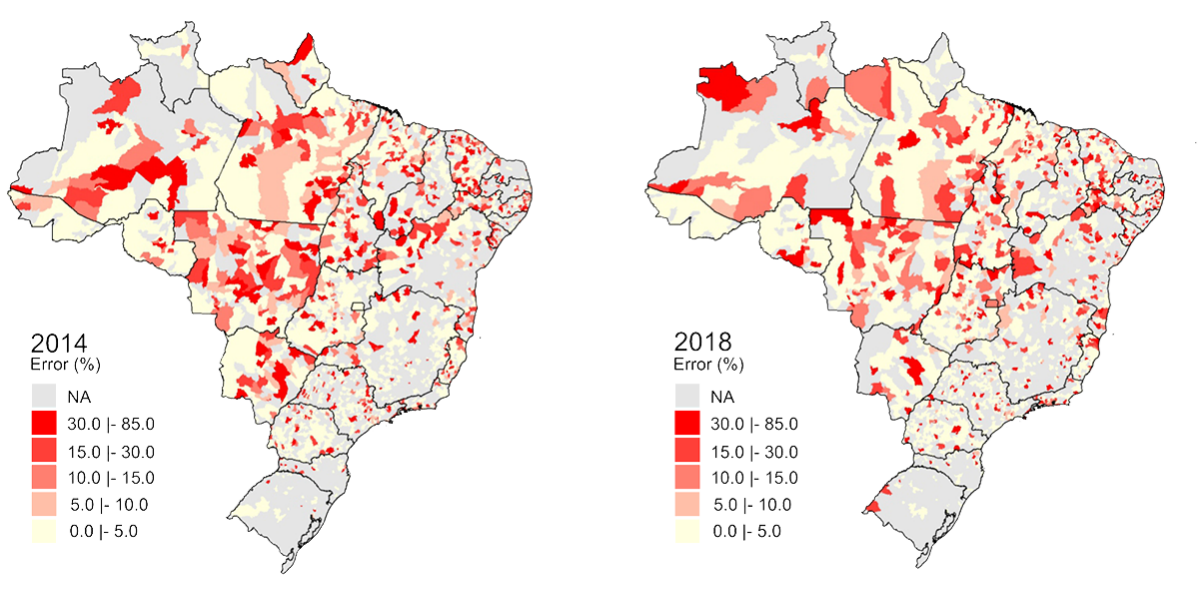
Geographic leprosy misclassification distribution in Brazilian municipalities. NA: not available.

Figure 6 shows that leprosy misclassification exhibited a certain degree of homogeneity throughout Brazil from 2014 to 2018, which does not raise a suspicion of its correlation with leprosy’s incidence. Notably, some municipalities had discrepancy rates reaching up to 80%, although in general this error rate appears to have decreased over time. Unlike other Brazilian states, Amazonas (in 2018) presented an unreasonable increase in the error rate, especially considering that these municipalities had virtually no notification of the disease in 2014.

### AI Model to Support Clinical Diagnosis

**Figure 7:**
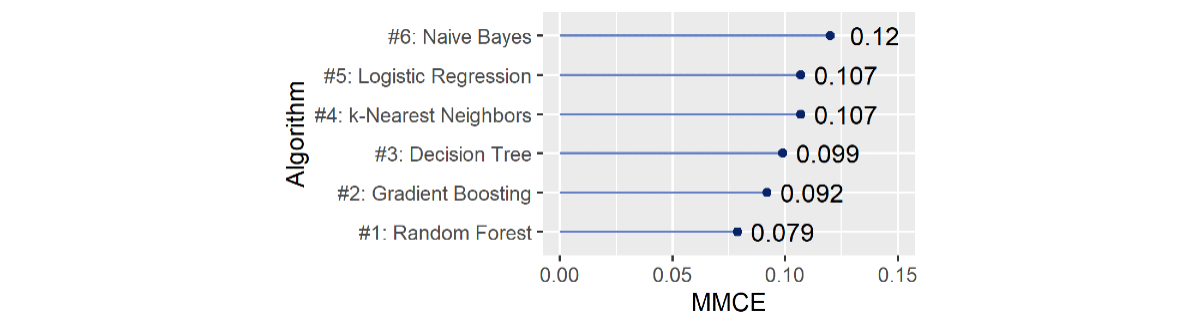
Comparison of algorithms according to the mean of misclassification error (MMCE).

Starting from the assumption that the error of clinical diagnosis is properly characterized, it was possible to develop a support model based on AI. The RF algorithm presented the smallest mean of misclassification error compared with the other algorithms using 10-fold cross-validation with default hyperparameters considered in the mlr R package [29] (Figure 7).

We next sought to determine if AI can assist in choosing the correct treatment for leprosy. Since the Brazilian Ministry of Health and the WHO suggest patterns (an algorithm) to classify the disease, our group was able to develop this new strategy to help control leprosy. A different model for each Brazilian state was used to improve the prediction in the remaining states; that is, a cross-validation strategy was applied to avoid overfitting by training in one state but testing in others. The number of lesions, incidence, and affected nerves were among the most important variables in the three best models (Mato Grosso, Rio Grande do Sul, and Paraná).

**Figure 8:**
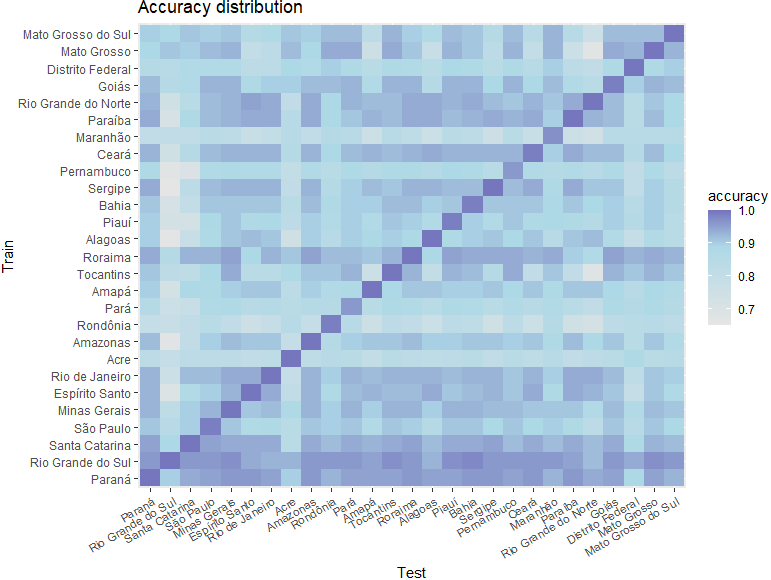
Quality in the classification of leprosy cases by artificial intelligence models in Brazilian states.

Figure 8 shows a heatmap of the quality of all models for each state of Brazil. The blue scale represents the accuracy of the models in each testing situation. The Y axis denotes the states used to build the models (training dataset) and the X axis indicates the states where the models were applied (testing dataset).

Table 3 shows the importance of the three best models (Mato Grosso, Rio Grande do Sul, and Paraná) used in development of the app. These models showed some variability in the importance of each variable. In the Mato Grosso model, the most important variables were the number of affected nerves, number of skin lesions, and incidence. In the Rio Grande do Sul model, the main variables were number of skin lesions and number of affected nerves. Finally, in the Paraná model, the most important variables were the number of skin lesions and bacilloscopy. “Gender” represented less than 2% of importance in all three models, whereas “number of skin lesions” represented more than 23% importance in all models.

**Table 3:** Importance (in percent) of each variable utilized in the models that represent the highest accuracy.

Variable	Meaning	Mato Grosso model	Rio Grande do Sul model	Paraná model
INCIDÊNCIA	Incidence	15.0	9.5	6.1
NU_IDADE_N	Age	9.3	8.1	5.5
CS_SEXO	Gender	1.6	1.7	1.9
CS_RACA	Race	2.7	6.9	1.2
CS_ESCOL_N	Educational level	4.7	6.4	3.4
NU_LESOES	Number of skin lesions	23.6	37.0	41.9
AVALIA_N	Grade of disability	4.4	4.2	5.0
BACILOSCOP	Bacilloscopy	6.1	4.5	23.7
CONTREG	Number of household contacts	5.1	5.9	2.8
NERVOSAFET	Number of affected nerves	27.5	15.8	8.5

Table 4 shows the quality of the AI model applied to the differential diagnoses of paucibacillary and multibacillary leprosy in 26,546 cases. We considered multibacillary as the reference. Some measures such as accuracy (proportion of multibacillary and paucibacillary cases correctly classified), sensitivity (proportion of correctly classified cases given that they were truly multibacillary), specificity (proportion of correctly classified cases given that were truly paucibacillary), positive predictive value (proportion of multibacillary cases given a positive classification by the model), and negative predictive value (proportion of paucibacillary cases given a negative classification by the model) are presented for each model.

**Table 4:** Quality of the artificial intelligence model applied to the differential diagnosis of paucibacillary and multibacillary leprosy.

Quality parameter	Mato Grosso model	Rio Grande do Sul model	Paraná model
Accuracy	0.970	0.812	0.929
Sensitivity	0.926	0.977	0.877
Specificity	0.812	0.218	0.919
PPV^a^	0.936	0.803	0.972
NPV^b^	0.786	0.740	0.698

^a^PPV: positive predictive value.

^b^NPV: negative predictive value.

## Discussion

### Relevance of Correct Operational Classification

This study analyzed the SINAN database from 2014 to 2018 considering the epidemiological, clinical, and sociodemographic context of patients diagnosed with leprosy in Brazil. In analyzing the frequency of inconsistencies in the SINAN database (Table 2), we observed that the main disagreement was operational classification (ie, multibacillary vs paucibacillary) that did not match the clinical form. These results highlight the importance of training health professionals to correctly identify the operational class and improving data collection. Information on the operational classification for a given case is essential to select the appropriate treatment scheme. Misclassification may harm both the patient and the health care system. A patient with paucibacillary leprosy receives treatment for 6 months, whereas a patient with multibacillary leprosy receives treatment for 12 months [17,30]. Therefore, a patient with paucibacillary leprosy misclassified as multibacillary leprosy would undergo 6 months of unnecessary treatment, burdening the health care system and increasing the risk of suffering from side effects during this time. By contrast, a patient with multibacillary leprosy misclassified as paucibacillary leprosy could be undertreated, requiring restarting the treatment that leads to spending more resources with potential subsequent disability rehabilitation.

According to Grossi et al [31], there is a strong tendency for health professionals to classify patients as having multibacillary leprosy, at least in Brazil. This trend, confirmed in our analysis of the SINAN database, seemed to be related to the absence of laboratory tests such as skin biopsy and slit-skin smear that provide security to professionals in decision making. Furthermore, Nobre et al [32] showed that the proportion of newly diagnosed leprosy cases that were cases of multibacillary leprosy increased by 11.6% during a study of cases reported in Brazil from 2001 to 2013. It is important to keep in mind that leprosy classification methodology may vary according to the diagnostic capacity of the center. In primary care settings, diagnosis and operational classification are mainly based on clinical findings, whereas in reference centers, confirmatory biopsies can be performed. Moreover, it is important to note that improper treatment can also lead to bacterial resistance [30-33].

### Relevance of the App

According to WHO goals for 2030, it is necessary to employ strategic methodologies to assist leprosy control. The use of AI is a novel method with potential to expand the capacities to diagnose diseases, especially those that are neglected. The use of AI therefore allows for obtaining higher coverage in the initial diagnosis process and facilitates the sharing of secure information, with the aim to expand and reach a larger number of health professionals [7].

We recognize the importance of a tool to improve the accuracy of insufficiently trained health professionals, especially in the most remote areas of Brazil. The app presented in this work proved to be a promising option to improve the coverage and scalability to the Brazilian health service regarding the choice of an appropriate treatment for leprosy [33]. The training of a physician requires considerable time when compared to computational diagnostic resources. In addition, the accessibility of this app in the hands of any professional via mobile or desktop devices offers scalability that traditional teaching methods cannot achieve [34]. Machine learning in standardized functions or decisions is expected to be much faster than the acquisition of general human knowledge. Therefore, our app would provide speed, scalability, and broadcasting to fight leprosy without compromising accuracy [35].

### Appropriate Data Collection for Leprosy Classification

Another relevant issue to mention involves problems in incorrectly filling out the reporting form. To fill out a SINAN form correctly, it is necessary to list the Madrid Classification and the treatment according to the operational classification. Therefore, a patient given a classification of indeterminate or tuberculoid has to receive treatment for paucibacillary leprosy, whereas a classification of borderline or lepromatous requires treatment for multibacillary leprosy.

According to the Brazilian Ministry of Health in 2017, a case with a positive BI result should be considered as multibacillary leprosy. However, doubts arise for cases that are considered to be borderline and close to the tuberculoid pole. Despite the difficulty of correct classification, these cases are generally considered to be multibacillary leprosy [17]. Another important point is related to the high rate of patients with missing or unknown data in the SINAN database. There were 77,628 patients with at least one data item missing, which represents 44% of the total. These data could be essential to improve the accuracy of machine-learning models in which performance is related to the amount of available clean data. This issue reinforces the necessity for developing a better approach for inputting data to the SINAN database as well as adopting good practices for validating these data. Lastly, the part of the form related to clinical aspects is filled out by the doctor, whereas the first part related to patient identification, in a general way, is filled out by the auxiliary staff. The state of Minas Gerais adopted this criterion; however, in other areas, other health professionals might complete the entire SINAN form, even the parts that are supposed to be completed by the physician. The Ministry of Health recommends that the physicians fill out the SINAN, make the notification and the diagnosis, as well as the documentation of successful treatment [17].

### Relevance of Using AI

In addition to the implementation of an algorithm that helps choose the correct therapy, the use of intelligent data collection devices would allow for higher quality and validation [36]. Such devices could be used to guarantee higher-quality data for future research and innovations regarding the improvement of health services as a whole, since poor-quality data is not limited to leprosy [37]. An algorithm is nothing more than a finite sequence of well-defined instructions that can be automated, usually to solve problems. The actions advocated by the WHO for the classification of leprosy to ensure appropriate treatment and cure of this disease represent an example of the algorithm itself. Considering this evident algorithm, our mission is to provide a statistically reliable emulation to aid in the diagnosis of leprosy. We have implemented a classification method based on RF, which is accessible using any and all devices with internet access.

Notably, the high accuracy (92.38%), sensitivity (93.97%), and specificity (87.09%) of this app provide a multiplatform method to support scalable characterization/classification for numerous other neglected diseases in remote communities in Brazil and worldwide.

### Limitations

As previously mentioned, SINAN is a platform launched to manage notifications from each Brazilian state. The records of this platform are obtained from assessments made by many health professionals with different levels of qualification. Thus, the quality of data depends on many factors, including (i) quality of the items requested by the forms and their correct interpretation, (ii) correct clinical assessment of the patient, and (iii) proper filling out of the forms. In addition, it is important to note that the possibility to add more items of information about serological and molecular integrated tests for leprosy diagnoses could undoubtedly improve the accuracy of the method, as we have done in our research group [13]. Lastly, the app is not currently available without an internet connection since the AI model is deposited in the RStudio cloud.

### Conclusions

The proposed app showed good accuracy to classify a case as paucibacillary or multibacillary leprosy by recognizing patterns in leprosy cases registered in the SINAN database. After validation, this app could be an essential tool to help health professionals make an accurate leprosy operational classification and decide which treatment to use for patients with paucibacillary or multibacillary leprosy considering reducing the likelihood of mistreatment. This study also highlights the importance of improving data collection methods given that prediction accuracy markedly increases with improved data quality.

## Abbreviations

AI: artificial intelligence
AUC: area under the receiver operating characteristic curve
BI: bacilloscopy index
FN: false negative
FP: false positive
FPR: false positive rate
NA: not applicable/not available
NTD: neglected tropical disease
RF: random forest
ROC: receiver operating characteristic
SINAN: Sistema de Informação Nacional de Agravos de Notificação (National Notifiable Diseases Information System)
SUS: Sistema Único de Saúde (Unified Health System)
TN: true negative
TP: true positive
TPR: true positive rate
WHO: World Health Organization
